# Assessment of junior doctors’ admission notes: do they follow what they learn?

**DOI:** 10.5116/ijme.58b1.4d7e

**Published:** 2017-03-11

**Authors:** Rashid A. Barnawi, Abdulaziz M. Ghurab, Hassan K. Balubaid, Sultan S. Alfaer, Kamal A. Hanbazazah, Mohammed F. Bukhari, Omayma A. Hamed, Talal M. Bakhsh

**Affiliations:** 1 Faculty of Medicine, King Abdulaziz University, Saudi Arabia; 2 Quality and Academic Accreditation Unit, Medical Education Department, Faculty of Medicine, King Abdulaziz University, Saudi Arabia; 3 Department of Surgery, Faculty of Medicine, King Abdulaziz University, Saudi Arabia

**Keywords:** Admission notes assessment, history-taking, junior doctors, admission notes completeness, physical examination, Saudi Arabia

## Abstract

**Objectives:**

To assess the completeness of history-taking and physical-examination notes of junior doctors at King Abdulaziz University Hospital per the approach they learned in medical school.

**Methods:**

In this retrospective study, we reviewed 860 admission notes written by 269 junior doctors (interns and residents) in an academic tertiary-care medical centre in Jeddah, Saudi Arabia, over a two-month period. Notes were evaluated for completeness using a checklist developed with reference to relevant medical textbooks. The checklist included 32 items related to history-taking and physical examination. Based on the review of the notes, checklist items were evaluated as complete, incomplete, not present, or not applicable according to set criteria. Data were analysed and summarised for information on the frequency and relative frequency of these types.

**Results:**

The history items varied in completeness. At the high end, asking about chief complaint and duration, associated symptoms, aggravating and relieving factors, and conducting systemic review were marked ‘complete’ in 74.2%, 81.7%, 80.4%, and 79.7% of notes, respectively. At the low end, asking about previous episodes, allergies, medications, and family history were complete in 5.3%, 1.9%, 4.8%, and 2.9% of notes, respectively. All physical examination items were poorly documented, especially breast examination, which was ‘not present’ in 95.8% of the notes.

**Conclusions:**

Junior doctors’ history and physical-examination notes are often incomplete and do not follow the approach taught in medical school. The reasons for this must be studied via focus-group discussions with junior doctors.

## Introduction

Medical history-taking and physical examination are essential tools in the practice of medicine.^1^ Complete and accurate history and physical-examination notes are vital for proper diagnosis and treatment.^2^ They are also crucial for the completeness of the medical record, which is a fundamental tool in patient care in the hospital setting.^3^ For these reasons, medical students in their clinical years are taught to take a standard comprehensive history and perform a thorough physical examination for all patients at the time of admission.^4^^,^^5^ Junior doctors who initially encounter and assess patients at the time of admission and write initial assessment notes for those patients are accordingly expected to follow that comprehensive approach.

Several published studies have aimed to assess the completeness and quality of medical records^6^^-^^9^ as well as the history-taking and physical-examination skills of junior doctors.^4^^,^^10^^-^^12^ These studies, conducted at national and local levels, concluded that the records they evaluated lacked essential data on the medical history and physical examination of the patients, and that junior doctors’ history-taking and physical-examination notes were incomplete (and, by inference, that their note-taking skills were inadequate). In a single-blinded observational study, Sandeep^4^ found that junior doctors (medical postgraduate year [PGY]-1 interns and PGY-2 residents) in a New York City teaching hospital had poor history-taking and physical-examination skills and, moreover, that there were discrepancies between the observed histories and examinations and the documented notes, as doctors documented examinations they had not actually performed. In a retrospective study, Oliver et al.^10^ investigated the hypothesis that junior doctors’ examination skills are deteriorating by assessing the admission records of patients admitted to Wellington Hospital in New Zealand over four decades. They concluded that there had been a deterioration in documentation, implying a deterioration in junior doctors’ physical-examination skills.

At King Abdulaziz University Hospital (KAUH) in Saudi Arabia, we have detected multiple deficiencies in the completeness of junior doctors’ (interns’ and residents’) history-taking and physical-examination notes as entered into the hospital’s electronic medical record health information system. On that basis, we speculated that the interns and residents acting as admissions clerks do not follow the standard approach taught to medical students - namely, taking a comprehensive medical history, performing a complete physical examination, and writing complete admission notes. No previous study has investigated the note-taking performance of interns and residents about what they were taught in medical school.

Given the importance of producing complete patient notes and following a standard, comprehensive approach in taking histories and performing physical examinations, this study aimed to assess the completeness of the histories and physical-examination notes taken by junior doctors at KAUH about the approach they learned in medical school.

## Methods

### Study design and participants

This retrospective study reviewed the admission notes of patients admitted over a two-month period (1 December 2014 to 31 January 2015) to KAUH, an educational hospital at King Abdulaziz University (KAU) in Jeddah, Saudi Arabia.

A sample of 860 admission notes was gathered by sampling admission notes for all patients admitted to KAUH for the first time during the two-month period. The notes were written by 269 interns and residents. An ‘intern’ is a recent medical school graduate receiving compulsory supervised training who is not yet licensed; a ‘resident’ is a fully licensed medical school graduate undergoing on-the-job training in a residency program. Admission notes were selected from the gynaecology (n = 130), surgery (n = 256), internal medicine (n = 210), and paediatrics (n = 160) departments as well as from the coronary care unit (CCU) (n = 104). Of the patients in the sample, those who were admitted more than once during the two-month period were identified, and only the first admission note was used for evaluation. In compliance with KAUH policies, data were collected after obtaining ethical approval from the Research Ethics Committee of the KAU Faculty of Medicine.

### Data collection methods

Data were collected via a checklist constructed to evaluate interns’ and residents’ history-taking and physical-examination notes in terms of the standard approach medical students at KAU are taught to follow. The checklist consisted of 32 history and physical-examination items that should be included in the standard histories and physical examinations of new patients. The items were obtained from textbooks on history-taking and physical examination used by KAU medical students.^13^^-^^16^ After it was created, the checklist was reviewed by several teachers in the Faculty of Medicine, including those from the departments of gynaecology, internal medicine, paediatrics, surgery, ophthalmology, and otorhinolaryngology (ENT). It was also reviewed by two expert medical educators. The checklist included items for the patient’s personal data (medical record number, gender, admission date, age, department, and admission diagnosis), followed by the 32 history and physical-examination items (20 history items and 12 physical examination items; see the Appendix).

### Procedure

We reviewed interns’ and residents’ notes through the hospital’s electronic medical record health information system. The checklist was filled in by the authors. During the review of the interns’ and residents’ notes, each item in the checklist was marked ‘complete’, ‘incomplete’, ‘not present’, or ‘not applicable’ (N/A) based on the completeness criteria (see appendix). An item was marked ‘complete’ if the notes fulfilled all criteria, ‘incomplete’ if one or more of the indicated criteria were missing, and ‘not present’ if the item was not mentioned in the notes at all. This ensured that evaluations would be accurate and consistent among the reviewers. The non-applicability of some items resulted from factors such as age, gender, and diagnosis (e.g. menstrual history in a male patient or musculoskeletal examination in a patient admitted as a case of myocardial infarction). Therefore, it was to some degree subject to the reviewer’s interpretation.

### Data analysis

The answers on the checklist were converted into codes, which were entered into SPSS version 23. The data were then analysed for frequency and relative frequency and were summarised. All N/A cases were excluded from the summary to present a clearer picture of the completeness of the documentation of each item.

## Results

Table 1 lists all history items, each with its sample size after excluding N/A cases. The table shows the percentages of complete, incomplete, and not-present data for each item. Among the most frequently completed items were those asking about chief complaint and duration, associated symptoms, aggravating and relieving factors, and systemic review items (74.2%, 81.7%, 80.4%, and 79.7% of the notes, respectively).

**Table 1:** Completeness of history-taking items

History Item	Complete N (%)	Incomplete N (%)	Not present N (%)
Asking about chief complaint and duration (n = 860)	638 (74.2)	188 (21.9)	34 (3.9)
Asking about associated symptoms (n = 860)	703 (81.7)	12 (1.4)	145 (16.9)
Asking about aggravating and relieving factors (n = 843)	678 (80.4)	11 (1.3)	154 (18.3)
Asking about previous episodes (n = 797)	46 (5.3)	1 (0.1)	750 (94.1)
Systemic review (n = 860)	685 (79.7)	15 (1.7)	160 (18.6)
Asking about allergies (n = 860)	16 (1.9)	803 (93.4)	41 (4.8)
Asking about past medical history (n = 860)	452 (52.9)	328 (38.1)	80 (9.3)
Asking about past surgeries (n = 860)	596 (69.3)	172 (20.0)	92 (10.7)
Asking about social history (n = 860)	569 (66.2)	208 (24.2)	83 (9.7)
Asking about family history (n = 860)	25 (2.9)	7 (0.8)	828 (96.3)
Asking about medications (n = 860)	41 (4.8)	21 (2.4)	798 (92.8)
Asking about previous transfusions (n = 860)	511 (59.4)	3 (0.3)	346 (40.2)
Asking about perinatal history (n = 134)	20 (14.9)	11 (8.2)	103 (76.9)
Asking about nutritional history (n = 157)	13 (8.3)	10 (6.4)	134 (85.4)
Asking about developmental history (n = 142)	13 (9.2)	9 (6.3)	120 (84.5)
Asking about immunisation (n = 156)	21 (13.5)	6 (3.8)	129 (82.7)
Asking about past gynaecological procedures (n = 124)	14 (11.3)	2 (1.6)	108 (87.1)
Asking about menstrual history (n = 116)	7 (6.0)	11 (9.5)	98 (84.5)
Asking about sexual history (n = 92)	3 (3.3)	-	89 (96.7)
Asking about contraceptive history (n = 109)	1 (0.9)	-	108 (99.1)

The least frequently completed items were those asking about previous episodes, allergies, medications, family history, sexual history, and contraceptive history (in 5.3%, 1.9%, 4.8%, 2.9%, 3.3%, and 0.9% of the notes, respectively; these items were ‘not present’ in most cases, except for ‘allergies’, which was ‘incomplete’ in most cases). The other history items were also, to varying degrees, inadequately documented in the notes where they were applicable, as shown in the table.

The completeness of each physical examination item and the sample size after excluding N/A cases are shown in Table 2. All physical examination items were poorly documented in the notes. The five physical examination items that are applicable in all cases and must be performed on every new patient (general, heart, respiratory, abdomen, and central nervous system examinations) were incomplete in most of the notes (92.4%, 96.9%, 96.5%, 96.2%, and 92.1%, respectively). Breast examination documentation was especially inadequate. It was not complete in any of the notes and was not present at all in most notes.  

**Table 2:** Completeness of physical examination items

Physical examination item	Complete N (%)	Incomplete N (%)	Not present N (%)
General examination (n = 860)	41 (4.8)	795 (92.4)	24 (2.8)
Heart examination (n = 860)	2 (0.2)	833 (96.9)	25 (2.9)
Respiratory examination (n = 860)	6 (0.7)	830 (96.5)	24 (2.8)
Abdominal examination (n = 860)	8 (0.9)	826 (96.2)	26 (2.9)
Central nervous system examination (n = 860)	36 (4.2)	792 (92.1)	32 (3.7)
Head and neck examination (n = 196)	39 (19.9)	133 (67.9)	24 (12.2)
Musculoskeletal examination (n = 116)	13 (11.2)	95 (81.9)	8 (6.9)
Lower limb examination (n = 179)	21 (11.7)	9 (5.0)	149 (83.2)
ENT (ear, nose, and throat) examination (n = 86)	10 (11.6)	57 (66.3)	19 (22.1)
Ophthalmic examination (n = 25)	2 (8.0)	6 (24.0)	17 (68.0)
Vaginal and pelvic examination (n = 131)	3 (2.3)	107 (81.7)	21 (16.0)
Breast examination (n = 24)	-	1 (4.2)	23 (95.8)

## Discussion

This study’s results imply that junior doctors at KAUH rarely follow the standard comprehensive approach they learned in medical school, and that their admission notes are incomplete, as evidenced by the observed inadequacy of documentation for most history and physical-examination items.^4^

As shown in Table 1, the extent of this inadequacy in certain areas - especially gathering information on previous episodes, allergies, medications, and family history - is remarkable and urgently needs to be addressed. Notes about previous episodes, medications and family history were mostly not present at all, though it is well known that genetic inheritance plays a major role in the development of various chronic diseases and cancers,^7^ and that medication history is important for detecting drug-related pathologies and drug-related changes in clinical signs.^17^ Information about patient allergies was present in most notes but was mostly incomplete (93.4%) since only drug allergies, not food allergies, were generally documented (see Appendix). On the other hand, notes about chief complaint and duration, associated symptoms, aggravating and relieving factors, and systemic review was adequate in most cases, implying that interns and residents are aware of the important role of these items in reaching a diagnosis.^7^ Nevertheless, there is much room for improvement and better note writing.

The deficiencies in the physical-examination notes were even more pronounced.^4^^,^^10^ Although interns and residents are taught that general, heart, respiratory, abdominal, and central nervous system examinations should be performed for every new hospital admission; they were inadequately documented in most cases. In an unexpected finding, other examination notes that are not expected to be performed for every hospital admission - such as head and neck, musculoskeletal, and ENT examinations - were more adequately written, though still very deficient. We have no explanation for this observation. Breast examinations were performed in almost none of the 19 breast disease admissions, of which 13 were breast cancer cases (the most frequently encountered surgical admission during the two-month period). In fact, breast cancer is the most common cancer in females worldwide and accounts for 22% of all new cancers among women in Saudi Arabia.^18^ Therefore, breast examinations in particular should be performed carefully and thoroughly.

There are several possible reasons for these results. First, interns and residents might intentionally omit details they consider unnecessary and time-consuming. They might also argue that omitting these details will not affect patient care. This argument is invalid, we think since there was a pronounced deficiency in specific history items that are necessary for addressing certain kinds of cases, such as menstrual history in gynaecological cases and vaccination history in paediatric cases. This also applies to breast disease cases, where interns or residents did not perform complete breast examinations in even one case. Moreover, interns and residents have been trained for years to take detailed histories and perform thorough physical examinations; they have had every opportunity to learn that following this approach offers the best chance of identifying any medical issues that warrant attention.^4^ However, we should not ignore the various factors - such as busy workloads - that could cause them to adopt contrary perspectives. Other possible explanations include a lack of the requisite knowledge or skills, negligence, or the increased availability of more accurate specialised diagnostic equipment, which might overshadow the use of clinical skills to gather diagnostic information.^10^

The first step toward addressing this problematic behaviour among interns and residents is to educate them about the importance of writing complete patient notes. Deficiencies can lead to errors that undermine the quality of healthcare and pose risks to patients’ health. In contrast, comprehensive clinical notes will lead to better and more accurate dissemination of information among healthcare providers and ensure the early detection of changes in patients’ health.^3^ Emphasis should be placed on the value of proper history-taking and physical examinations, and their superiority over diagnostic equipment for diagnosing some medical conditions.^2^ Junior doctors should also recognise their influence as role models for medical students.^19^ Poor history-taking and physical examinations constitute poor role modelling, which has a detrimental effect on students.^20^ However, when medical students observe the thorough approach they learned in school applied in practice, they recognise its importance and effectiveness.^21^ Senior doctors should also fulfil their roles as role models for interns and residents (junior doctors) by showing commitment in their own practice and encouraging juniors to follow the rules. They should emphasise the need for integrity in every aspect of medical practice, including proper history-taking and physical examination. Enhanced senior supervision and assessment of the history-taking and physical-examination activities and skills of interns and residents, including formative feedback, could have a positive effect.^10^ There is also evidence that improved undergraduate curricula, especially bedside teaching, and increased supervision of new doctors can improve clinical skills.^10^ One method for overcoming a lack of knowledge would be to distribute standardised criteria for writing admission notes to all interns and residents to ensure they are aware of them and to emphasise the importance of following them. Pre-printed assessment sheets based on these criteria might also improve the quality of notes,^9^and decrease ambiguity and the inadvertent omission of data.^3^ Improving the structure of the hospital’s electronic medical record system and mandating that detailed notes be produced are suggested as well.^22^ Moreover, the yearly auditing of admission notes may encourage interns and residents to maintain complete notes.^8^

The limitations of this study include the fact that it did not verify the specific reasons why junior doctors do not follow a comprehensive approach. Understanding why this occurs is very important if we are to find solutions. Furthermore, this study did not determine whether admission notes written by residents are more scant or defective than those written by interns, or vice versa, due to difficulties in retrieving information on the precise status of note writers through the electronic medical record system. If residents’ notes had been found to be more defective than interns’ notes, this might have helped determine whether the hospital environment played a role in reducing the quality of documentation. Another limitation is that we did not address confounders that might affect junior doctors’ performance, such as staff shortages. It is possible that busy workloads could negatively affect their clinical skills.^10^ It is worth noting that not all interns and residents included in this study attended the KAU Faculty of Medicine, and this study did not compare the notes of those who studied at KAU with those who did not. However, the concept of following a standard comprehensive approach is taught to medical students universally;^5^^,^^23^ any variations should be minor and should not significantly change our findings.

Future research should examine the specific reasons why these interns and residents did not follow the standard comprehensive approach. In particular, conducting focus-group discussions with interns and residents might help determine whether the causes include, for example, a lack of knowledge and skills, negligence, or a belief that what is taught to medical students is not entirely applicable in practice.

## Conclusions

This retrospective study at KAUH found that junior doctors’ admission notes are incomplete and that they do not take comprehensive histories for new patients or perform thorough physical examinations in the way they were taught in medical school. We outlined several possible reasons for this; further study and verification are needed to find solutions. Focus-group discussions with junior doctors could be the first step. It is also crucial to educate interns and residents about the importance of writing complete notes. Enhancing the supervision of junior doctors’ clinical skills, giving formative feedback, improving undergraduate bedside teaching, and using pre-printed assessment sheets are other possible ways to improve junior doctors’ clinical skills and the quality of the resulting documentation.

### Acknowledgements

We wish to thank the clinicians and teachers who kindly gave their time to participate in this study and shared their experiences with us. In particular, we acknowledge Dr Hind Fallatah (Department of Internal Medicine, King Abdulaziz University Hospital, Jeddah, Saudi Arabia) and Dr Heidi Al-Wassia (Department of Paediatrics, King Abdulaziz University Hospital, Jeddah, Saudi Arabia) for their valuable efforts in reviewing this study.

### Conflict of Interest

The authors declare that they have no conflict of interest. 

## Supplementary material

Supplementary file 1 Completeness criteria for history-taking and physical-examination notes
