# Supplementary material for: Assessment of junior doctors’ admission notes: do they follow what they learn?
Source: Int J Med Educ. 2017 Mar 11;8:79–87. doi: 10.5116/ijme.58b1.4d7e (PMC5357543; doi:10.5116/ijme.58b1.4d7e)
Supplement: Supplementary file 1 — Completeness criteria for history-taking and physical-examination notes [file ijme-8-79-S1.pdf]

Appendix

Completeness criteria for history-taking and physical-examination notes

History

History item	Criteria for completeness<br>(all of the following must be mentioned in the notes to mark the item 'complete')
Asking about chief complaint and duration	1. Chief complaint<br>2. Duration
Asking about associated symptoms	1. Positive symptoms<br>2. Important negatives<br>(e.g. 'no associated shortness of breath' in case of chest pain without shortness of breath)
Asking about aggravating and relieving factors	1. Aggravating factors<br>2. Relieving factors
Previous episodes	- Yes or no?<br>If yes:<br>1. How many?<br>2. When?
Asking about systemic review	1. Cardiovascular<br>2. Respiratory<br>3. CNS<br>4. Gastrointestinal<br>5. Genitourinary<br>6. Endocrine<br>7. Rheumatological<br>8. Haematological<br>9. Dermatological
Asking about allergies	1. Drug allergy<br>2. Food allergy
Asking about past medical history	- Does patient have established chronic diagnosis?<br>If yes:<br>3. What is the disease?<br>4. Duration?
Asking about past surgical history	- Yes or no?<br>If yes:<br>5. What type of surgery?<br>6. When?
Asking about social history	1. Smoking<br>2. Alcohol<br>3. Drug abuse<br>4. Occupation
Asking about family history	1. Similar disease in the family<br>2. Other chronic diseases in the family<br>3. Financial status<br>In case of paediatrics patient only:<br>4. Who lives with the child and takes care of them?
Asking about medications	- Is the patient taking medications chronically?<br>If yes:<br>1. What are the medications?<br>2. How long has the patient been taking them?

Asking about transfusion	- Yes or no?<br>If yes:<br>1. How many times?<br>2. When?<br>3. Any complications?
Asking about perinatal history (antenatal, intranatal, and postnatal)<br>(applies to paediatrics patients only)	Prenatal history:<br>Is the baby premature?<br>1. Any prenatal baby complications? (e.g. bleeding, diseases, radiation)<br>Intranatal history:<br>1. Apgar score<br>2. Spontaneous vaginal delivery or delivered by caesarean section? If delivered by caesarean section, what was the reason?<br>Postnatal history:<br>1. Birth weight?<br>2. ICU admission/needed ventilation?<br>Conditions, e.g. jaundice, cyanosis, disease?
Asking about nutritional history<br>(applies to paediatrics patients only)	1. Formula fed, breastfed, or both?<br>- If breastfed:<br>a. Frequency?<br>b. Duration?<br>- If formula fed:<br>c. Type of formula?<br>d. Total daily intake?<br>e. Frequency?<br>f. Duration?<br>2. Age of introduction of solid food, and what kind of food was introduced?<br>3. Age of weaning<br>4. Type of diet: normal family diet or special diet?
Asking about immunisation<br>(applies to paediatrics patients only)	- Has the child taken all vaccines for his or her age?<br>If not, what is the reason?
Asking about developmental history<br>(applies to paediatrics patients only)	1. Fine motor skills according to age<br>2. Gross motor skills according to age<br>3. Social according to age<br>4. Language and hearing according to age
Asking about menstrual history	1. Age of menarche<br>2. Last menstrual period<br>3. Regularity<br>4. Length<br>5. Quantity of discharge<br>6. Associated symptoms
Asking about sexual history	1. Dyspareunia<br>2. Post-coital bleeding<br>3. Sexually transmitted diseases
Asking about contraception history	1. Type<br>2. Duration<br>3. Any complications?
Asking about past gynaecological procedures history<br>(applies to gynaecology patients only)	- Yes or no?<br>If yes:<br>1. What type of procedure? (e.g. D&C, pap smear, pelvic ultrasound, colposcopy)<br>2. When?

Physical examination

Physical examination item	Criteria for completeness<br>(all of the following must be mentioned in the notes to mark the item 'complete')
General examination	1. Appearance (well or ill)<br>2. Body built (cachectic or overweight?)<br>3. Abnormal discoloration (jaundice, pallor, or cyanosis)<br>4. Distress<br>5. Deformities<br>6. Vital signs
Heart examination	1. Inspection<br>2. Palpation<br>3. Auscultation
Nervous system examination	1. Mental status examination<br>2. Motor examination:<br>a. Inspection<br>b. Palpation<br>c. Tone<br>d. Power<br>e. Reflexes<br>Sensory, cerebellum, and cranial nerves examination are performed only in neurological cases
Respiratory examination	1. Inspection<br>2. Palpation<br>3. Percussion<br>4. Auscultation
Abdominal examination.	1. Inspection<br>2. Palpation<br>3. Percussion<br>4. Auscultation
Head and neck examination	1. Thyroid<br>a. Inspection<br>b. Palpation<br>c. Auscultation<br>2. Lymph nodes in the head and neck<br>a. Palpation
Musculoskeletal examination	1. Inspection<br>2. Palpation<br>3. Power<br>4. Special test depending on the joint
Lower limb examination<br>(peripheral vascular examination)	1. Inspection<br>2. Palpation<br>3. Auscultation
Vaginal and pelvic examination	1. Inspection<br>2. Palpation and/or speculum exam
Breast examination	1. Inspection<br>2. Palpation

ENT examination	1. Ear<br>a. Tympanic membrane<br>b. External auditory canal<br>2. Nose<br>a. Mucosa<br>b. Turbinate<br>c. Septum<br>d. Secretions<br>3. Mouth<br>a. Mucosa<br>b. Teeth<br>c. Hard and soft palate<br>d. Tonsils<br>e. Oropharynx
Ophthalmic examination	1. Pupil examination<br>2. Extraocular muscles movements<br>3. Anterior segment (anterior chamber, iris, and lens)<br>4. Fundal examination.

ICU: intensive care unit; D&C: dilation and curettage; ENT: ear, nose, and throat; CNS: central nervous system
